# Account of Two Cases of Poisoning by Lobelia Inflata

**Published:** 1850-07

**Authors:** 


*Account of two Cases of Poisoning by Lobelia Inflata.* By T. Wood, M. D., Cincinnati, Ohio.

I am inclined to look on quacks as a very useful class of practitioners. They are experimenters, and, strange as it may seem, are seldom in want of voluntary subjects, to swallow any medicine they may fancy to have mirarculous virtues; and they seldom fail to persevere in its use, till its effects are known on every constitution, age, and sex, as well as all the diseases that are found in the catalogue of human ills. It is a subject of regret that their experiments cannot be witnessed by competent observers, and the results accurately recorded for the benefit of science; but this is generally out of the question. The enlightened observer is mostly a man having human sympathy, and cannot silently witness suffering that he can prevent or allay. The quack gives his medicine with his mind in a state of mystified confidence that it will do good, and has not enough of knowledge to distinguish between the effects of his nostrum, and the disease that he designs it to cure, and therefore, can look with indifference on a scene that would shock a man of science. If the scientific practitioner were to witness, without disapprobation, one of the many deaths that occur from empirical experimenting, he would be indicted for manslaughter; while the very man who gave the fatal dose, would be acquitted by a jury, on the plea of ignorance. But to return. If a regular record could be kept of the effects of all the nostrums used and vended by quacks in every case, much useful information would be gained by the regular profession; and the many lives that are sacrificed, would not all be lost in vain, but their martyrdom would be a legacy of infinite value to the wise and prudent patrons of the legitimate healing art. But while I regard quacks as conferring great favors on a large portion of the human family, who are fortunate enough to escape from being their victims, I cannot help feeling astonishment at the strange infatuation they exercise over so many persons, and over those who are distinguished for good sense and intelligence, on all subjects, except that of their own health.

Many of our most valuable remedies have been introduced to the notice of the regular profession by quacks, and often at a fearful sacrifice of life, before their true virtues were known; and perhaps none have been introduced at a greater cost of life, than that of lobelia inflata. I have seen many cases of injury by its use as an emetic, but the two following will be sufficient to illustrate its poisonous properties.

Case 1st. In the summer of 1843, I was requested by the friends of J. Johnston, of Mt. Pleasant, Jefferson county, Ohio, to make a post mortem examination of his remains, and if possible, discover to them the cause of his sudden and unexpected death. During the course of the examination, the following facts were elicited:

Mr. Johnston, a carpenter, had been a healthy hard working man; but from some cause had been troubled for the last three years of his life, with an eruption about the nose, which he was anxious to get rid of, more from its unsightly appearance than from any pain that he had experienced, or danger that he apprehended from its presence. He had taken the advice of several physicians, but none had given him permanent relief; and he had finally concluded that it was his fate to bear his affliction, without a hope of cure. But while he was trying to reconcile his mind to his gloomy destiny, his hopes were suddenly rekindled by a *steam doctor*, who had lately stepped into the professional ranks by a "short cut," and had made his appearance in the village. This man promised to eradicate every vestige of the disease from his nose, by one "course of medicine, to be applied by the most approved principles of Sam'l Thompson's system; and as a further inducement to Mr. J. to give it a trial, told him that if he would commence on Saturday night, after quitting his work, he might go through it on Sunday, and on Monday morning resume his labors, a renovated if not transfigured man, free from all his bodily ills.

Mr. J. was allured by the quack's promises, and determined to test his skill; and on Saturday night about six o'clock, he took the first dose of his medicine, and in thirty-three hours he breathed no more.

The steamer was present at the examination, and gave in substance the following statement of his treatment: "Four table spoonfuls of the Tinc. of lobelia were given, at intervals of fifteen minutes between each dose, with a view to induce vomiting. By the time the last dose was taken, the patient complained of extreme sickness of stomach, entire prostration of strength, and a frothy phlegm was rising into his mouth, nearly strangling him, but no vomiting could be induced. A table spoonful of saleratus was then dissolved in a teacupful of water, and the patient made to swallow it to 'kill the effects of the lobelia,' as Thompson directs. Shortly after this, he was seized with intense burning pain in the stomach, and was given a cup of vinegar to destroy the saleratus." This caused such violent eructations, and hiccough, as to alarm Mr. J.'s family, and Drs. Hamilton and Flanner were sent for, but it was too late—their efforts could not save him.

The dissection showed a softened mucous membrane, but the most striking peculiarity was the great distention of the stomach and bowels, by the gas liberated by the action of the vinegar on the potash. As the walls of the abdomen were cut through, the stomach and bowels burst from their confinement, with a force that seemed to threaten a rupture of their coats. This enormous distention existed in the stomach, extended through the small intestines, and terminated at the ileo-coccal valve. The colon throughout its whole extent, was contracted so much as nearly to obliterate its cavity—presenting the appearance of a solid cord, about the thickness of a man's finger, and it had nothing in it but a small quantity of mucus.

After the examination was completed, I was requested to make a statement of my opinion of the cause of death, in the council room, to the assembled citizens of the village. The steamer was present, and with the effrontery characteristic of an ignorant, unprincipled charlatan, answered, and explained all that I said, much to the satisfaction of himself and his friends. When I stated my belief that the man lost his life by the steamer's treatment, he declared that the patient was killed by the calomel doctors who had taken him out of his hands, and that if his course of medicine had been gone through with, the man would be still alive.

I endeavored to explain impartially to the audience, the effects of lobelia on the system, when not thrown from the stomach by emesis, and the corrosive effects of saleratus on its coats in such large doses, and lastly, the action of the vinegar on the alkali with the disengagement of gas. When I read from the United States Dispensatory, the evidence of the poisonous nature of lobelia, the steamer read from the work of Dr. Curtis, of Cincinnati, the proof that it was an innocent and harmless plant. As to saleratus, he said it could not hurt any person, for it was eaten every day in our bread and cakes—and to say that vinegar was poison, "it was too ridiculous to deserve any comment from him."

I left Mt. Pleasant convinced of the fallacy of an attempt to reason with a popular audience, on a medical subject; for the steamer made them believe that he was a persecuted man, and their voice was in his favor. His practice, however, was very limited, and his next patient was his own wife, whose death was about as sudden as that of Mr. J.'s, but no examination was had of her case.

The steamer finally abandoned his profession, probably disgusted with the uncertainties of medicine; and the last I knew of him, he was selling candy and pea nuts in the city of Covington.

It appeared from the testimony of his friends, that Mr. Johnston had never vomited in his life, his stomach having been peculiarly insensible to the action of emetics.

Case 2d. On the 13th of June, 1849, I was called to see John Goffern, who had taken three teaspoonfuls of powdered *lobelia*, under the direction of a steamer who has a store on Main street, above Eighth, Cincinnati.

When I arrived, (about two hours after his taking his first dose,) the patient was covered with a cold clammy sweat—pulse 40, and very feeble—skin blue, particularly about the head and face—abdomen very much distended with fluids—and the whole muscular system relaxed, so that he had no control over the sphincter of the anus to retain injected fluids; and his abdomen felt like a loose sack filled with water—destitute of muscular fibres. The pupils of the eyes were contracted, and the action of the brain much stupefied. He had an insatiate thirst, and complained of intense agony in the stomach. I requested the advice of Dr Latta, who concurred with me in the opinion that the use of the stomach pump was necessary to save his life. It was used by Dr. L. with success, giving relief in a short time.

The steamer had prescribed the lobelia to cure the ague, but, although the man had experienced the full effects of the medicine, he had a chill on the day following its administration.

The ague soon yielded to the proper use of quinine, and Mr. Goffern has no desire to try *lobelia* again.—*Western Lancet.*

